# Clinical effect of Anwei Qingyou recipe on peptic ulcer and its effect on the levels of EGF and PGE2: A retrospective study

**DOI:** 10.1097/MD.0000000000030898

**Published:** 2022-10-07

**Authors:** Guoxin Bai, Junyu Yi, Yuanyuan Yu, Hongxing Du

**Affiliations:** a Department of Traditional Chinese Medicine, Jinhua Jindong District Hospital of Traditional Chinese Medicine, Jinhua, Zhejiang Province, P.R. China; b Department of Traditional Chinese Medicine, Ningbo Fenghua Xiabang Hospital of traditional Chinese Medicine, Ningbo, Zhejiang Province, P.R. China; c Department of Otorhinolaryngology, Jinhua Jindong District Hospital of Traditional Chinese Medicine, Jinhua, Zhejiang Province, P.R. China.

**Keywords:** Anwei Qingyou recipe, peptic ulcer, triple therapy, ulcer healing

## Abstract

To investigate the effect of Anwei Qingyou formula on the clinical treatment of patients with peptic ulcer (PU) and its effect on the levels of epidermal growth factor (EGF) and prostaglandin E2 (PGE2).

**Methodology:**

Medical records of 83 patients with PU, treated in our hospital from January 2020 to January 2021, were retrospectively analyzed. Among them, 40 patients received conventional triple therapy (scheme I), that is, oral omeprazole, clarithromycin and amoxicillin, twice a day, and 43 patients received conventional triple therapy + Anwei Qingyou formula, taken orally twice a day (scheme II). The improvement of clinical symptoms, the quality of ulcer healing, clinical effectiveness and recurrence rate were analyzed after 4 weeks of treatment. Patients were followed up for six months.

**Results:**

After treatment with corresponding regimen, the total clinical effective rate of scheme II was 97.57% (42/43), which was significantly higher than 82.50% (33/40) of scheme I. Six-month follow-up results showed that the recurrence rate in scheme II patients was 4.65% (2/43), which was significantly lower than 20.00% (8/40) in the scheme I group (χ ^2^ = 5.479, 4.607, all *P* < .05). After one course of treatment, the levels of serum EGF and PGE2 in scheme II group were higher than those in scheme I group (*P* < .05).

**Conclusion:**

In combination with the conventional western medicine treatment, Anwei Qingyou formula administration in PU patients effectively improves the overall control of the disease and therapeutic effectiveness.

## 1. Introduction

Peptic ulcer (PU) is a common digestive system disease in clinical diagnosis and treatment. The clinical symptoms of patients include dull, swelling or tingling pain in the epigastric region near the heart pit of the upper abdomen that is often accompanied by different degrees of acid reflux noise, nausea, vomiting, loss of appetite. Gastric distention and other related upper gastrointestinal discomfort symptoms have a negative impact of patients’ quality of life.^[1,2]^ PU is characterized by high incidence rate, long course of disease, and tendency to relapse. If not effectively and timely controlled, PU can become aggravated, and lead to serious complications such as perforation, ulcer bleeding, and even gastric cancer, which endangers the patient’s health and affects the quality of life.^[3]^ The specific pathogenesis of PU is not clear. In recent years, the incidence rate of PU has been increasing. At present, studies have shown that PU is mainly caused by Helicobacter pylori infection and excessive gastric acid secretion, and Helicobacter pylori infection is responsible for the disease recurrence.^[4,5]^ In addition, recent studies have shown that motilin and gastrin play an important role in the pathogenesis of PU. Additionally, heredity, mood, diet, and environment seem to be closely related to the occurrence of PU.^[6]^At this stage, the commonly used regimen for the treatment of PU patients is gastric mucosal protectants or proton pump acid inhibitors + antibiotics. However, this treatment requires patients to take medication for a long time. Patient compliance, therefore, will have a significant impact on the overall curative effect. Moreover, long-term medication may lead to drug resistance, and the recurrence rate is relatively high.^[7]^ In recent years, the research on the clinical treatment of PU patients with traditional Chinese medicine and integrated traditional Chinese and Western medicine has been increasing and has shown good application effect. However, the optimal treatment scheme with best effectiveness and safety has not been determined. The main goal of this study is to assess clinical effect and value of traditional Chinese medicine, Anwei Qingyou formula in combination with the conventional western medicine treatment on PU patients.

## 2. Materials and Methods

Medical records of 83 PU patients (47 males and 36 females), treated in our hospital from January 2020 to January 2021, were selected for retrospective analysis. The ethics committee of our hospital approved this study (Number: JDL21132, Date: 2021-10-20). Inclusion criteria were as follows: PU was definitely diagnosed by X-ray barium meal examination and gastroscopy; rapid urease test was HP-positive; relevant clinical diagnosis and treatment data were available. Exclusion criteria were as follows: Other gastrointestinal diseases, such as reflux esophagitis and gastric cancer; blood diseases, active central nervous system diseases, severe liver, and kidney dysfunction, etc; previous history of gastrointestinal surgery, serious PU complications and mental diseases; allergic constitution, allergic to drugs used in the study.

Patients were retrospectively divided into two groups, based on the type of therapy they received during the treatment. Patients that were treated with the conventional triple therapy comprised scheme I group. Patients, treated with the combination of the conventional triple therapy and Anwei Qingyou formula comprised scheme II group.

All processes of this study fully complied with the relevant rules and regulations of the medical ethics committee of our college.

Conventional triple therapy treatment scheme included: oral omeprazole enteric coated tablets (Shandong new era pharmaceutical industry, national drug approval Zi h20044871, specification: 10 mg * 28 tablets) 20 mg/time, twice a day; Oral clarithromycin tablets (Shanghai Abbott pharmaceutical, gyzz h20033044, specification: 250 mg * 8 tablets) 0.5 g/time, 2 times/day; Oral amoxicillin capsule (Sanjing Mingshui pharmaceutical industry of Harbin Pharmaceutical Group, gyzz h23023294, specification: 0.25 g * 50 capsules) 1 g/time, 2 times/day. Continuous medication for 4 weeks was a course of treatment.

Conventional triple therapy + Anwei Qingyou formula scheme included conventional triple therapy treatment scheme and Anwei Qingyou formula. The ingredients of the formula were as follows: Codonopsis pilosula 20g, Atractylodes macrocephala and galangal 15g each, Radix Paeoniae Alba, Radix Glycyrrhizae, Radix Astragali and areca 10g each. All medicines in the prescription were provided by the herbal medicine room of the hospital, and were decocted by the decocting machine in the traditional Chinese medicine pharmacy. When decocting to 200 mL, take the Medicine cocktail, decocted to 200 mL, was taken orally twice a day for 4 weeks.

Medical records of all included patients contained basic characteristics and relevant indicators that were collected after completing one course of treatment. The following indicators were analyzed: Total clinical efficacy: complete disappearance of relevant clinical symptoms and signs. Patient was considered recovered if gastroscopy showed completely healed ulcer surface. PU was considered significantly improved if the ulcer area seen by gastroscopy was reduced by ≥30% compared with that before treatment. PU was considered not improved or further aggravated if gastroscopy showed that the ulcer area was not significantly shortened or further increased.^[8]^ Total clinical efficacy was calculated as a ratio of (cured + improved)/total cases × 100% = total effective rate. Recurrence. After one course of treatment, all patients were followed up for 6 months, and the records of HP infection recurrence at the last follow-up were collected. Indicators of ulcer healing quality: 5 mL of morning fasting venous blood was collected before treatment and 1D after a course of treatment. After centrifugation, the levels of prostaglandin E2 (PGE2) and epidermal growth factor (EGF) were detected by enzyme-linked immunosorbent assay.

Statistical analysis and processing were performed by SPSS 22.0 software. The measurement data were expressed as (± s) and processed by one-way ANOVA. The counting data was expressed as a percentage “%”, which is used for counting χ^2^. *P* < .05 was considered statistically significant.

## 3. Results

Medical records of 83 PU patients (47 males, 36 females, aged 24–69 yr) met the inclusion criteria and were retrospectively analyzed. Patients were divided into two groups based on the treatment regiments. Forty patients were treated with scheme I (conventional treatment) and 43 patients were treated with scheme II (conventional treatment + Anwei Qingyou formula) regiments. There was no significant difference between the two groups in gender, age, course of the disease and other related basic pathological characteristics (*P* > .05) (Table 1). After one course of treatment with the corresponding scheme, the total clinical effective rate of scheme II was higher than that of scheme I. The follow-up results showed that the recurrence rate of the disease in patients, treated with the scheme II regiment was lower than that of scheme I, and the difference was statistically significant (*P* < .05) (Table 2).

**Table 1:** Comparison of baseline data between the two groups.

Scheme	n	Sex ratio	Age (yr, ± s)	Course of disease (mo, ± s)	Ulcer diameter (cm, ± s)
		(Male*/*Female)			
Scheme I	40	24/16	24~68 (43.67 ± 11.73)	4~25 (14.15 ± 4.85)	0.42~1.38 (0.79 ± 0.20)
Scheme II	43	23/20	24~69 (45.81 ± 12.52)	5~27 (14.77 ± 5.28)	0.40~1.40 (0.81 ± 0.24)
*χ^2^/t*	-	0.358	0.801	0.554	0.467
*P*	-	.550	.425	.581	.642

**Table 2:** Comparison of total effective rate and recurrence rate between the two groups.

Scheme	n	Total efficacy				Relapse
		Recovery	Significant improvement	No improvement	Total effective rate	
Scheme I	40	14 (35.00)	19 (47.50)	7 (17.50)	82.50%	8 (20.00)
Scheme II	43	24 (55.81)	19 (41.86)	1 (2.33)	97.57%	2 (4.65)
*χ^2^*	-	-	-	-	5.479	4.607
*P*	-	-	-	-	.019	.032

There was no significant difference in the levels of serum EGF and PGE2 between the two groups before treatment (*P* > .05). After one course of the treatment with the corresponding scheme, the two index levels of the two groups were significantly improved. At the same time, the index levels of scheme II were significantly higher than those of scheme I (*P* < .05) (Table 3).

**Table 3:** Comparison of serum EGF, PGE2 levels between the two groups.

Scheme	n	EGF (ng/mL)		PGE_2_ (PS/mL)	
		Before treatment	After treatment	Before treatment	After treatment
Scheme I	40	0.192 ± 0.030	0.454 ± 0.040	2.28 ± 0.74	4.80 ± 0.79
Scheme II	43	0.197 ± 0.029	0.697 ± 0.039	2.44 ± 0.68	7.61 ± 0.65
*t*	-	0.184	27.902	1.007	11.151
*P*	-	.855	<.001	.317	<.001

## 4. Discussion

The results of this retrospective study show that the combination of the traditional Chinese medicine Anwei Qingyou and the conventional western medicine treatment can improve the remission effect of clinical symptoms and signs in PU patients, obtain more ideal clinical therapeutic effectiveness and reduce the risk of recurrence. Combined treatment (scheme I) significantly increased the total effective rate of clinical treatment (97.57%) compared with that of conventional therapy alone (82.50%). The recurrence rate at six months after the treatment was 4.65% for scheme I patients, significantly lower than 20.00% recurrence rate of scheme I patients, *P* < .05. Our results are consistent with the previous reports showing beneficial therapeutic effect of traditional Chinese medicine in patients with PU. In the study of Wang Guochen et al,^[9]^ 36 patients with Helicobacter pylori-positive PU were treated with triple therapy + traditional Chinese medicine, Qingyou Hewei decoction, for 1 week. The total effective rate of clinical treatment in this group was as high as 97.22%. After treatment, the PU symptom score of patients was significantly lower than that before treatment and lower than that of formula I. The eradication rate of H.pylori in the patient group was as high as 94.44%. There were statistically significant differences between the groups.

Modern pharmacological reports suggest that the components of Anwei Qingyou formula used in our study may have beneficial therapeutic effect on patients with PU. For example, Astragalus membranaceus has anti-inflammatory effects, promotes ulcer wound healing, and helps to better alleviate gastric mucosal injury, Codonopsis pilosula can effectively promote the vitality of gastrointestinal tract, and some have anti-ulcer effect, Radix Paeoniae Alba has bacteriostatic and anti-inflammatory effects, Atractylodes macrocephala can effectively regulate intestinal activity, areca nut can effectively promote intestinal peristalsis, and Galangal has strong anti-ulcer effect.^[10]^ The simultaneous use of all these drugs is helpful to improve the recovery effect of gastric mucosal barrier. In this study, the levels of serum EGF and PGE2 in patients treated with scheme II were significantly higher than those before treatment, and were significantly higher than those in scheme I. Our results suggest that the use of Anwei Qingyou formula plays a significant role in promoting ulcer wound healing. The results of animal experiments in the study by Lu BP et al^[11]^ showed that the levels of serum PGE2, EGF and NO in rats with gastric ulcer increased significantly after receiving MAO Dexi’s Anwei Qingyou Prescription. Xu Z^[12]^ showed that in PU patients treated with Anwei Qingyou formula, the ulcer area reduction was significantly greater than in patients treated with conventional western medicine only. Moreover, the radical cure rate of HP was significantly higher, and the levels of serum EGF, PGE2 and NO after treatment were significantly higher in patients that received Anwei Qingyou formula. Together with these reports, our results suggest that the application of Anwei Qingyou formula plays an important role in improving the radical cure rate of HP, promoting ulcer wound healing and improving the healing quality, thus potentially improving the prognosis of patients.

This study has limitations. It is a retrospective, single center study with small sample size and follow-up time of only 6 months. Further multi-center studies with larger sample sizes, more observational indexes and longer follow-ups are needed.

## 5. Conclusion

The application of Anwei Qingyou formula in the clinical treatment of PU can significantly improve the ulcer wound healing effect and clinical effectiveness, and may contribute to providing treatment reference for clinicians.

## Author contributions

GB conceived and designed the study. JY, YY and HD collected the data and performed the analysis. GB was involved in the writing of the manuscript and is responsible for the integrity of the study. All authors have read and approved the final manuscript.

**Conceptualization:** Guoxin Bai, Yuanyuan Yu, Hongxing Du.

**Investigation:** Guoxin Bai.

**Methodology:** Junyu Yi, Yuanyuan Yu, Hongxing Du.

**Validation:** Guoxin Bai, Yuanyuan Yu, Hongxing Du.

**Writing – original draft:** Yuanyuan Yu, Hongxing Du.

**Writing – review & editing:** Guoxin Bai, Yuanyuan Yu, Hongxing Du.
